# Modeling and Testing of the Sandwich Composite Manhole Cover Designed for Pedestrian Networks

**DOI:** 10.3390/ma12071114

**Published:** 2019-04-03

**Authors:** Calin Itu, Camelia Cerbu, Teofil-Florin Galatanu

**Affiliations:** 1 Department of Mechanical Engineering, Faculty of Mechanical Engineering, Transilvania University of Brasov, B-dul Eroilor, No. 29, 500036 Brasov, Romania; calinitu@unitbv.ro; 2 Department of Civil Engineering, Faculty of Civil Engineering, Transilvania University of Brasov, B-dul Eroilor, No. 29, 500036 Brasov, Romania; galatanu.teofil@unitbv.ro

**Keywords:** composite material, glass fibers, bending, manhole cover, pedestrian networks

## Abstract

This research concerning the topic, pursues the design, manufacturing, analysis and testing of the manhole cover that may be used in pedestrian networks. Although there are certain commercially available manhole covers made of glass-reinforced composites, there are a few papers published related to the modelling, simulation and mechanical testing of such parts. Herein, the manhole cover is made of the sandwich composite. The novelty of this kind of cover is to use an oriented strand board (OSB) as the core between two sides containing layers, which are reinforced with glass fibers. The OSB core leads to the increase of the stiffness-weight ratio. The paper describes the materials corresponding to the layers of the composite cover, geometry of the cover, technology used to manufacture the bending specimens and cover tested. Specimens made of materials that correspond to each layer of the cover, are tested in bending in order to determine their mechanical properties (flexural strength and flexural modulus). Bending tests and testing of the cover are also described. The composite manhole cover is also analysed by the finite element method to obtain the state of stresses and strains. The strains of the manhole cover are experimentally measured by using the tensometric method. Finally, comparing the strains with the strains experimentally measured validates the numerical simulation model.

## 1. Introduction

In the field of civil engineering, it is no longer a novelty to use both plastics or glass fibers reinforced plastics (GFRP) to manufacture pipelines used for: Potable water supply and transportation; chemical installations for transporting the corrosive fluids; sewage systems for wastewater [1]. In such GFRP pipes, thermoset resins (polyester, vinyl-ester, epoxy) are usually used as a matrix material to bond glass fibers [1,2,3,4]. Flexible composite pipes made of high-temperature polythene (PE-RT) reinforced with aramid fibers are thermo-stable with a temperature that is lower than 95 °C and with the internal pressure that is lower than 4 MPa [5]. Fiber reinforced cementitious composites and composites based on thermoplastic rejects reinforced with foundry sand and steel, are also used to manufacture prefabricated elements like manhole covers for water and sewage networks or for pedestrian traffic [6,7].

In recent years, composite manhole covers and composite tank covers have been widely used for access to district heating systems, fluid transport systems, sewage systems and geothermal applications [8,9]. In such environments, there are some corrosion problems that lead to the replacing of the manhole covers made of traditional materials (steel, cast steel, reinforced concrete) with new generations that are made of composite materials as GFRP. There are many advantages of the manhole covers made of composite materials with respect to the metallic manhole covers: These provide high strength-weight and stiffness-weight ratios; are lightweight and therefore, they are easy to handle by one person; high resistance to chemical agents (corrosion); they are electrical insulators and provide significant reduction in heat transfer and thus, they protect the pedestrians from the underground environment; they are less noisy than cast iron covers under pedestrian or vehicle traffic [2,3,8,9].

Last but not least, composite materials are not easily recyclable. Thus, it eliminates the risk of theft because they have no resale value. Consequently, it also eliminates the risk of serious accidents and injuries caused by missing manhole covers. Another advantage of using GFRP to manufacture covers is that such materials offer little interference with the wireless communication signals in the case of protective applications of electrical switching for smart grid technology.

It is known that GFRP used to manufacture the manhole covers are sensitive to environmental conditions (moisture, thermal cycles, UV rays) because the properties of the polymeric matrix change under long-term environmental effects [1,2,3,10,11,12]. In case of the vinyl-ester resin, some properties (glass transition temperature, degree of crosslinking, tensile strength) increases with respect to exposure time to outdoor conditions up to 12 months. Then, small declines were recorded for longer exposure times [2].

Composite materials that are based on polyester or on vinyl-ester resins randomly reinforced with glass fibers show a good stability of the mechanical properties after a prolonged immersion in water [10,11,12]. After 9264 h (about 13 months) of immersion in water, small decreases were recorded for both flexural modulus E and flexural strength (with 5% and with 3.2%, respectively) with respect to the case using the composite materials based on Heliopol 9431 ATX polyester resin [10]. After the same duration of immersion in water, the flexural modulus and flexural strength decreases (with 6.7% and with 12.2%, respectively) with respect to the case of the composite materials based on Atlac 582 vinyl-ester [10]. In the same conditions of immersion, GFRP based on LY554 epoxy recorded decreases in the mechanical properties: The flexural modulus E experienced a decrease of 22% while flexural strength experienced a decrease of 31.25% [10]. These results show that Heliopol 9431 ATX polyester resin may be recommended to manufacture pipes or manhole covers used for sewage systems. Degradations of both fiber-matrix interface and matrix are the causes of the decrease of mechanical properties (flexural strength, modulus of elasticity) in case of the polymeric matrix reinforced both with glass fibers and wood flour [11] and in case of the polymeric composite laminates [12] under hydrothermal fatigue.

There is a study showing that replacing all of the steel hatch covers of a bulk carrier with composite covers reduces the weight by 44.32% which leads to many benefits including fuel saving [8]. Moreover, the foremost hatch cover was strengthened to withstand a 50% higher load than the standard because it is easier to manufacture complex geometry in case of the composite materials [8]. In case of the manhole covers underneath pavement surface, under heavy traffic loads, a novel material was proposed that is a combination of two-layers: one layer made of modified asphalt concrete as the base and a layer made of dense-graded asphalt concrete [13].

Another paper [9] shows that the anisotropy of the composite materials and joints between segments must be taken into account in the design and the failure analysis of composite tank covers built of repetitive two-wave segments with flanges overlapping one another. It is known that the joints based on polymeric composite materials reinforced with different kinds of fibers are sensitive under environmental conditions [14,15].

The subject of this study consists of using a novel sandwich laminated composite material as an alternative material for manhole covers used in pedestrian networks. This kind of sandwich composite material consists of: Sheet layers made of polyester resin reinforced with glass fibers; and an oriented strand board (OSB) used as the core. An OSB board is a commercial one. The novelty of this kind of manhole cover is the use of an OSB in the core of the laminated composite material that provides a higher stiffness-weight ratio for the manhole covers. Moreover, wood particleboard and polyester composite materials reinforced with wood fibers exhibit large deformations before failure [16]. The composite manhole cover with an OSB core has the advantage of a reduced cost of the raw material because an OSB core is cheaper than cores made of glass fiber reinforced polymeric resin.

In order to use this novel sandwich composite to manufacture the manhole cover that could be used for water and sewage networks or for pedestrian traffic, it is required to analyse the stresses and strains developed in the mechanically loaded manhole cover. Consequently, the main purpose of this research is the finite element analysis (FEA) of the stresses and strains that take place in the manhole cover made of such sandwich composite material. For this purpose, it is necessary to achieve some objectives. In order to accurately define the properties of each layer in the finite element model, the first objective is to determine the mechanical properties corresponding to the materials of each layer by using bending tests on specimens made of such kinds of materials. Then, in order to validate the finite element model, the values of strains corresponding to certain points of the numerical model are compared with the corresponding strains measured by using strain gages with the tensometric method. 

## 2. Materials and Methods 

### 2.1. Materials

#### 2.1.1. Composite Layers

**Figure 1:**
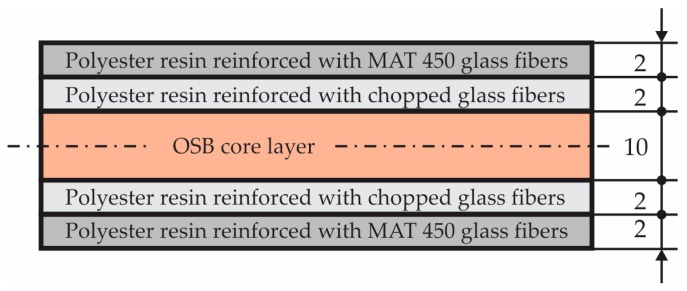
Structure of the sandwich composite material used to manufacture the manhole cover.

The structure of the sandwich composite material used to manufacture the manhole cover is shown in Figure 1. The sandwich composite contains five layers in cross-section: One core layer made of OSB4 Superfinish board (manufactured by Kronospan Romania SRL, Brașov, Romania) having thickness equal to 10 mm; two layer sheets made of sub-layers of Heliopol 9431 ATX polyester resin reinforced with MAT 450; two layers made of Heliopol 9431 ATX polyester resin reinforced with short chopped E-glass fibers whose length was max. 5 mm. 

OSB4 Superfinish boards are load-bearing boards for use in humid conditions and are wood-based products made from thin veneer strands that are bonded together with a synthetic resin. OSB4 boards are heavy duty load-bearing boards for the use in humid conditions. The mats made of thin veneer strands are pressed into panels by means of high temperature and pressure in the course of an uninterrupted continuous pressing process [17].

The material MAT is a fiberglass chopped strand mat (non-woven fabric) whose commercial name is EMC450 (Jiahe Taizhou Glass Fiber Co. Ltd., Taizhou, China) whose density is 450 g/m^2^ [18]. It is a non-woven E-glass fiber mat manufactured by spreading continuous filament roving of 50 mm in length randomly and uniformly in combination with polyester binder in powder. It is suitable for the applications of hand lay-up FRP moulding, such as: Various sheets and panels, boat hulls, corrosion resistant, vehicles [18].

Heliopol 9431 ATX polyester resin (manufactured by Helios) has the following physical properties: 700–1000 mPa⋅s–viscosity at 23 °C; 35–39% styrene content; 10–15 min. –gelling time; density 1100–1150 kg/m^3^; glass transition temperature 90–100 °C [19].

It is well-known that in the finite element analysis of a manhole cover, the mechanical properties of the materials corresponding to each layer must be defined. In order to determine the mechanical properties of the materials corresponding to each layer (Figure 1), the experimental tests were performed on standardized specimens according to the European standards (EN ISO 14125, EN 310) [20,21] regarding the shape and size of the specimens. Ten OSB bending specimens were cut from a commercial OSB4 board having 10 mm in thickness. In order to manufacture the composite specimens reinforced either with MAT450 or with short chopped E-glass fibers, it was first manufactured by two composite boards having dimensions 400 × 300 × 6.5 mm^3^. The content of the glass fibers is equal to 42 wt% in case of the composite made of polyester resin reinforced with MAT450. The content of the glass fibers is equal to 30 wt% in case of the composite made of polyester resin reinforced with chopped glass fibers. Then, ten bending specimens were cut from each kind of composite board reinforced with glass non-woven fabric MAT450 or with short chopped glass fibers, respectively. The specimens were subjected to the bending test by using the three point’s method.

#### 2.1.2. Geometry and Manufacturing of the Manhole Cover

**Figure 2:**
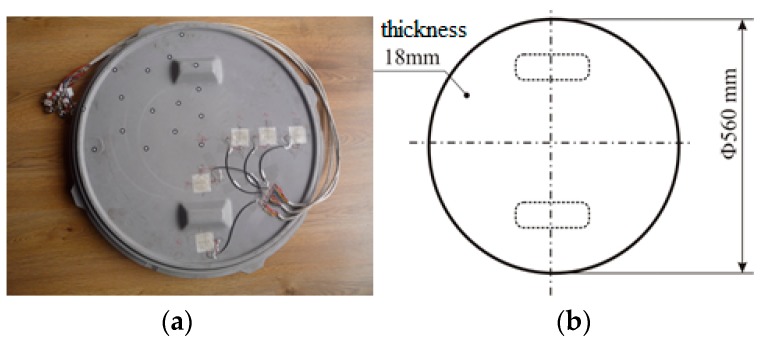
The manhole cover made of sandwich laminated composite material: (**a**) Photo; (**b**) sketch with main dimensions.

In Figure 2, the manhole cover that was tested is shown. The stresses and strains developed in the manhole cover were analysed by using the finite element method. This type of composite manhole cover is recommended to be used in pedestrian traffic applications or water networks. 

**Figure 3:**
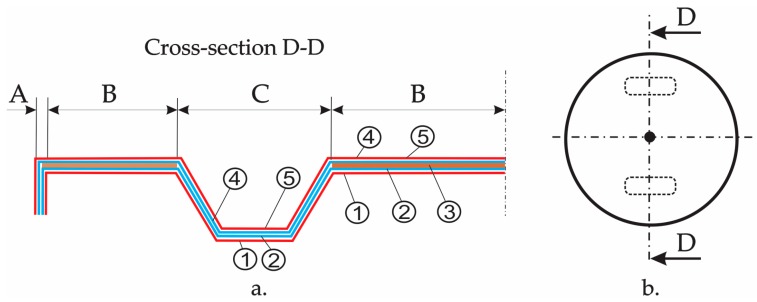
Schematic layout of the layers for the manhole cover (one to five are layers whose materials are shown in Table 1): (**a**) Layout of the layers corresponding to half of cross-section D-D of the manhole cover; (**b**) Top view of the manhole cover (handling areas are shown with dashed lines).

Figure 3, shows the schematic layout of the material layers of the composite manhole cover. In Figure 3, it is numbered with one to five the layers whose corresponding materials are shown in Table 1. According to Figure 3, half of the M-M section was divided into three areas as follows: Area A corresponds to the edge of the manhole cover; area B represents the horizontal area containing the core layer made of OSB; area C is the handling area. The geometry was changed with respect to the metallic manhole cover, in the handling area C and in area A (Figure 3) corresponding to the edge of the manhole cover, in order to fix the manhole cover on the support of the manhole cover that is embedded in in pavement.

The manufacturing process has a crucial influence on the mechanical behaviour of the structures made of composite materials [22]. The manhole cover involved in this study was provided by company SC Compozite SRL from Brasov (Romania). Herein, the manufacturing of the manhole cover is briefly described. The first, a thin layer of wax 827 (Axson Technologies) in paste form was applied on the mould that is the negative of the cover. After four hours of drying at room temperature (about 22 °C), the layers made of polyester 9431 ATX reinforced with glass non-woven fabric MAT 450 were placed on the mould (content of the glass fibers was 42 wt%). Then, the layers made of polyester 9431 ATX reinforced with chopped glass fibers (30 wt% of glass fibers), were placed on the mould. Before the polyester resin hardens, the OSB core (layer three from Figure 3) was placed on the mould after it has been cut in advance from a commercial OSB panel having 10 mm in thickness. Then, the next layers of the manhole cover in accordance with Figure 1 and Figure 3 are placed in the mould. Finally, the laminated composite manhole cover was kept for 48 h in the mould at about 22 °C. After removing it from the mould, it was kept in the same conditions until testing.

### 2.2. Mechanical Testing

#### 2.2.1. Coupon Tests

**Figure 4:**
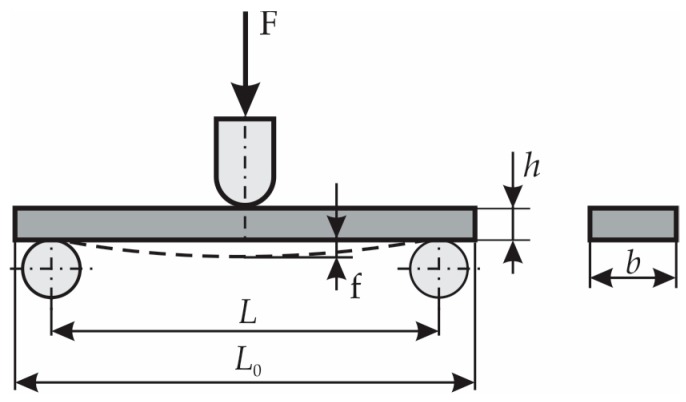
Bending test (three point’s method).

The bending test that use the three-point’s method was performed by using LLOYD LS-100 machine manufactured by the LLOYD Instruments (West Sussex, UK). The LLOYD LS-100 machine can be used for testing up to 100 kN. The scheme of loading is shown in Figure 4. The span L between the simple supports is shown in the last column of Table 1. The speed of loading was 1 mm/min in case of the composite materials reinforced with glass fibers (corresponding to the layers 1, 2, 4, 5 in Table 1). The speed was 4 mm/min in case of bending test conducted for OSB specimens.

All specimens tested have a parallelepiped shape. Their dimensions are shown in Table 1.

Force–deflection curves (F–f) have been obtained for each specimen tested in the bending load. 

The force-displacement curves (F–f) recorded in the bending tests were statistically processed and the mechanical properties were determined for each kind of material tested: Flexural strength that is the normal stress σ at maximum load; flexural modulus E that was determined on the linear portion of the force-displacement curve.

The flexural stress σ is computed by using Equation (1) [20,21]:

(1) $$\sigma =\frac{3}{2}\frac{FL}{bh^{2}},$$

where F is the external force applied; dimensions L,b,h are shown in Figure 4 and their corresponding values are shown in Table 1, in case of all materials tested.

The flexural modulus E is computed by using Equation (2) [20,21]:

(2) $$E=\frac{L^{3}}{4bh^{3}}\frac{\Delta F}{\Delta f},$$

where Δf represents the difference between the maximum deflections $f^{\prime \prime }$ and $f^{\prime }$ (Figure 4) measured at two different times (for example, standard [20] recommends $f^{\prime }=0.04$ and $f^{\prime \prime }=0.23$ in case of the involved composites corresponding to the layers 1, 2, 4 and 5); ΔF is the difference between the forces $F^{\prime \prime }$ and $F^{\prime }$ corresponding to the deflections $f^{\prime \prime }$ and $f^{\prime }$, respectively. In fact, ΔF/Δf is the slope of F−f curve located between points corresponding to $f^{\prime }$ and $f^{\prime \prime }$ deflections. In order to draw the stress-strain (σ−ε) curve corresponding to the bottommost cross-sectional points of the critical cross-section located at midpoint between the supports, the flexural stress σ is computed by using Equation (1) while the strain is calculated by using Equation (3):

(3) $$\varepsilon =\frac{6fh}{L^{2}}$$

#### 2.2.2. Testing of Manhole Cover

The measuring technique used to measure and record the strains that occurred in the manhole cover is based on the method of electro-resistive tensometry.

**Figure 5:**
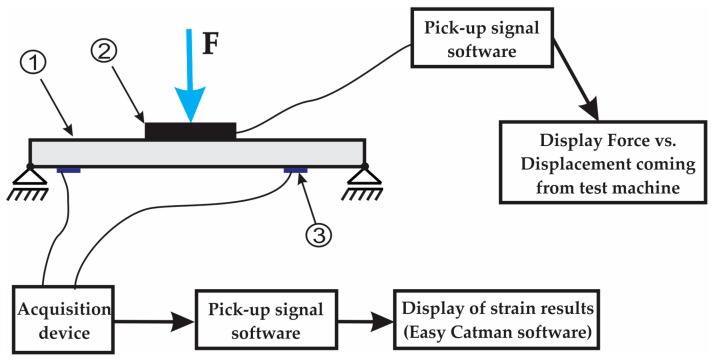
Scheme stand the test of laminated composite plate: 1-manhole cover tested; 2–Punch used to apply the force; 3–strain gauges.

**Figure 6:**
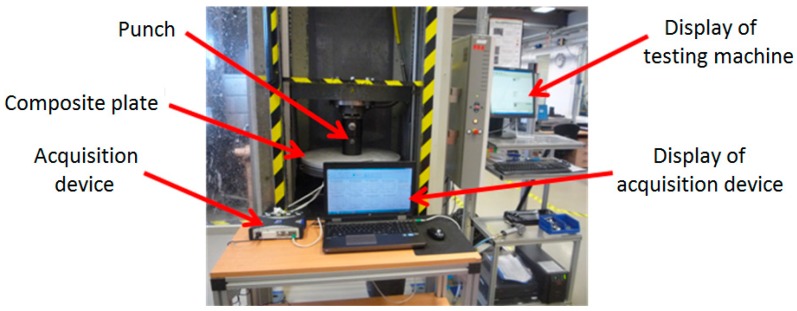
Photo of the experimental setup used to measure the strains developed in the composite manhole cover.

The scheme of the experimental stand used is shown in Figure 5. The external force F (coming from the vertical force actuator of the testing machine) is applied to the top side of the manhole cover one by means of the cylindrical punch denoted by two as shown in Figure 5. A photo of the experimental setup used in testing the manhole cover is shown in Figure 6. During the test, the compatible software of the machine displays the real-time curve of the force F related to the displacement of the vertical force actuator.

During the tests, the force applied on the composite manhole cover varied from 0 to 2000 N. Three tests were performed with three different loading speeds to see if the loading speed parameter has an influence on the structural behaviour of the manhole cover.

**Figure 7:**
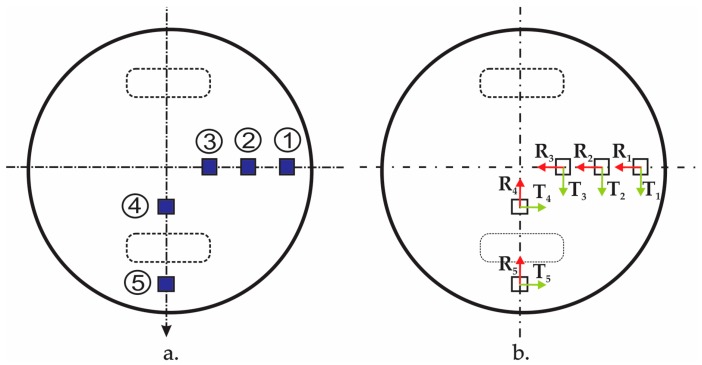
Strain gauges fixed on the bottom of the manhole cover: (**a**) Numbering and positions; (**b**) measuring directions.

In order to measure the strains developed at the bottom side of the manhole cover, five strain gauges are fixed on the bottom side of the composite manhole cover before testing, in the areas indicated in Figure 7a. In Figure 7b, the measuring directions of the strain gauges are indicated as follows: R indicates the radial direction; T indicates the tangential direction. Table 2 shows the positions of the strain gauges with respect to the centre of the manhole cover.

In order to measure the strains, quarter-bridge arrangements and strain gauges that can measure strains in two directions were used. The rectangular rosette gauges were manufactured by the Micro-Measurements Division (Raleigh, NC, USA). Their electrical resistance is 120.0 Ω ± 0.4% (24 °C).

The strains acquired by the strain gauges were transmitted into the Easy Catman software through the QuantumX MX840 Universal Measuring Amplifier (data acquisition device in Figure 6). QuantumX MX840 (manufactured by Hottinger HBM) has the frequency of 19.2 kHz and contains adapters for ¼ and ½ bridge strain gauges.

### 2.3. Finite Element Analysis of the Manhole Cover

The experimental data obtained from the bending tests were used to define the properties of the material corresponding to each layer of the numerical model of the manhole cover. The finite element analysis has been performed to obtain the stresses and strains developed in the manhole cover that was mechanically loaded in order to evaluate its strength performance.

**Figure 8:**
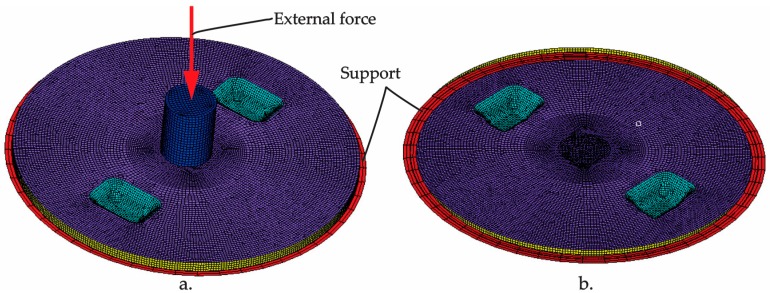
Finite element model of the manhole cover: (**a**) Top view; (**b**) bottom view.

The finite element analysis of the manhole cover was performed by using the commercial software ABAQUS. The finite element model of the manhole cover is shown in Figure 8.

The S4 shell element having rectangular shape, with four nodes located in corners, is used in the analysis. Such element is based on the theory of small and medium thick plates and can be used in the analysis of large deformations. This element has four corner nodes and each node has six degrees of freedom. The finite element model (Figure 8) contains 18104 elements and 18126 nodes (16370 nodes correspond to the cover, 250 nodes correspond to the support, 250 nodes correspond to the punch). 

The boundary conditions and the external load applied to the model are shown in Figure 8. The external force is applied progressively on punch (green colour) and is transmitted to the manhole cover through the contact area defined between the punch and the composite manhole cover. All freedom degrees were blocked for all nodes (250 nodes) corresponding to the support of the cover because it is embedded in the pavement. The contact of type node-to-surface was defined in the finite element model between the cover and punch and between the cover and support. 

The properties (Table 3) of the material defined in the finite element analysis are determined in the bending tests described in the previous section. The transverse contraction coefficient is defined in accordance with the scientific literature and with the previous published works by authors [23,24]. The composite layers randomly reinforced with chopped glass fibers or with MAT 450 glass fibers can be considered to be isotropic material at the macroscopic level due to the random reinforcement with fibers.

**Figure 9:**
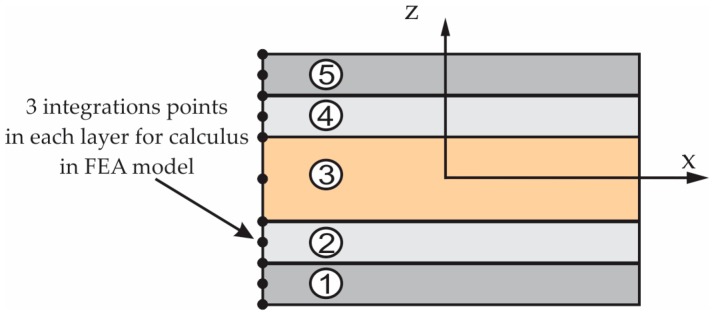
The integration points used for the composite layers of the manhole cover (materials corresponding to the layers 1–5 are shown in Figure 1).

Figure 9 shows the layers of the laminated composite material assigned to the part corresponding to the manhole cover, laminated material defined in FEA. The integration points (bottom, median and upper side of each layer) are also shown on the thickness of each layer. In the FEA model, each shell elements contain five layers that correspond to the layers of the cover and their integration points are shown in Figure 9. In this way, the perfect bonding between the layers of the cover is assumed.

## 3. Results

### 3.1. Results Obtained in Testing of the Materials

**Figure 10:**
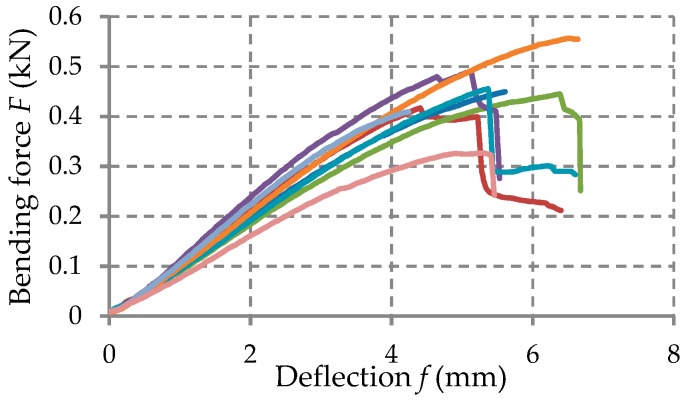
Force–deflection (F−δ) curves obtained in case of the OSB flexural specimens.

**Figure 11:**
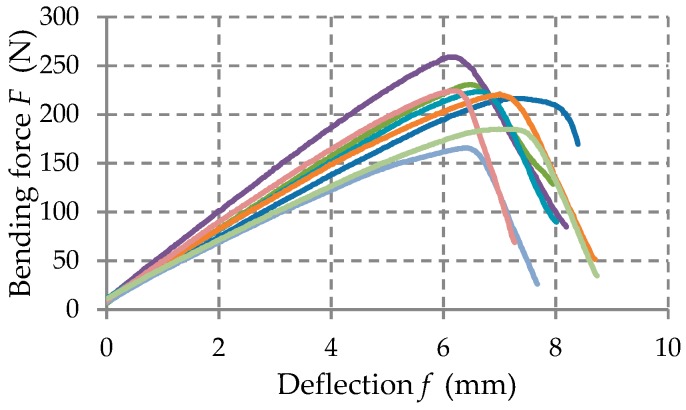
Force–deflection (F−δ) curves obtained in case of the specimens made of MAT 450 glass fibers/polyester resin.

**Figure 12:**
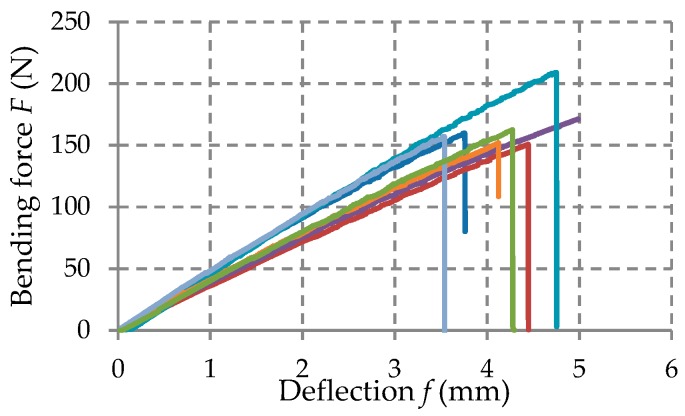
Force–deflection (F−δ) curves obtained in case of the specimens made of chopped glass fibers/polyester resin.

The force-deflection (F−δ) curves recorded in bending tests are shown in Figure 10, Figure 11 and Figure 12 for each set of certain material tested. Table 4 shows the average mechanical properties of the materials corresponding to each layer of the manhole cover structure.

### 3.2. Results Obtained from FEA

**Figure 13:**
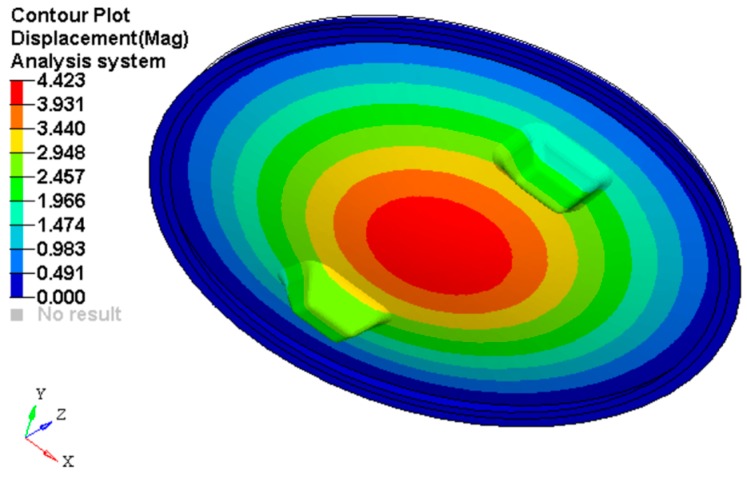
Plot of the displacements on the manhole cover (*F* = 2 kN).

Figure 13 shows the plot of the displacements obtained for 2000 N force applied on the top side of the punch. The maximum value of the displacement is 4.423 mm.

**Figure 14:**
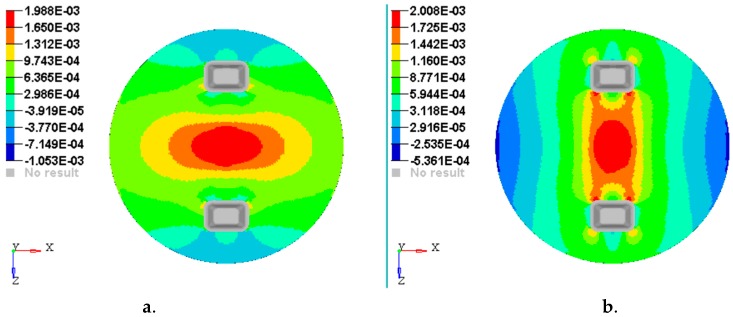
Plot of the strains on the bottom face of layer 1: (**a**) Strains $\varepsilon_{x}$ with respect to Ox axis; (**b**) strains $\varepsilon_{z}$ with respect to Oz axis.

**Figure 15:**
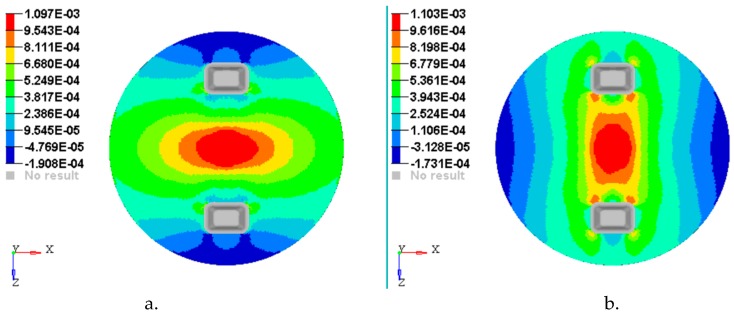
Plot of the strains on the interface between layer 2 and 3: (**a**) Strains $\varepsilon_{x}$ with respect to *Ox* axis; (**b**) strains $\varepsilon_{z}$ with respect to *Oz* axis.

**Figure 16:**
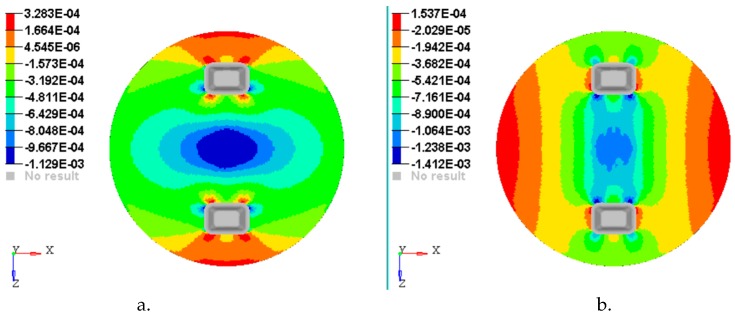
Plot of the strains on the interface between layer 3 & 4: (**a**) Strains $\varepsilon_{x}$ with respect to *Ox* axis; (**b)** Strains $\varepsilon_{z}$ with respect to *Oz* axis.

**Figure 17:**
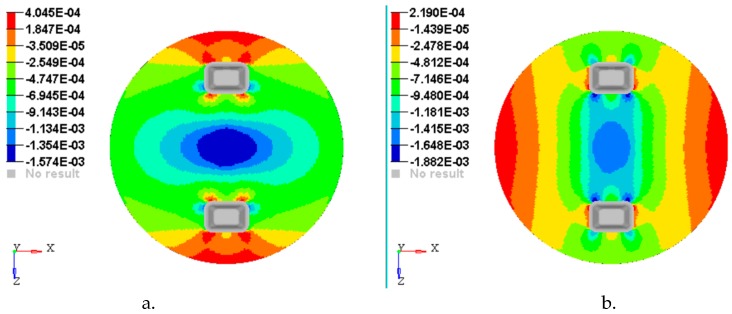
Plot strain on the interface between layer 4 and 5: (**a**) Strains $\varepsilon_{x}$ with respect to *Ox* axis; (**b**) strains $\varepsilon_{z}$ with respect to *Oz* axis.

**Figure 18:**
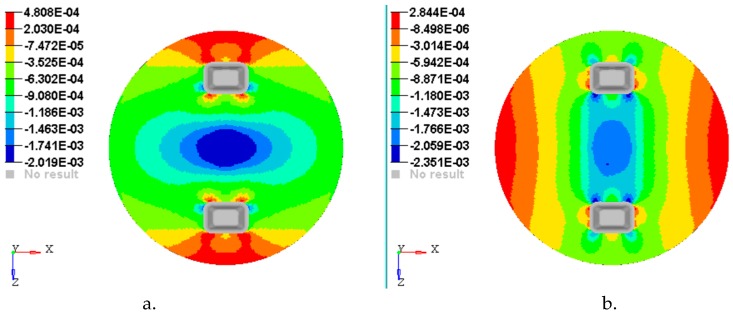
Plot strain on the upper face of layer 5: (**a**) Strains $\varepsilon_{x}$ with respect to *Ox* axis; (**b**) strains $\varepsilon_{z}$ with respect to *Oz* axis.

Figure 14, Figure 15, Figure 16, Figure 17 and Figure 18 show the plots of the strains developed in the bottom and upper side of each layer and the plot strains obtained at the interfaces between the two adjacent layers on x and z directions.

**Figure 19:**
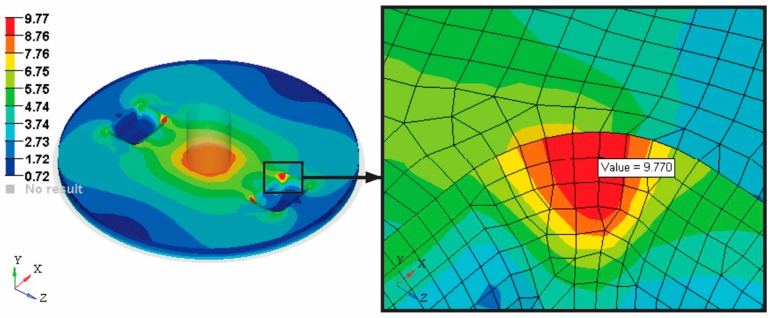
Plot of the Von Mises stress distribution on the manhole cover analysed.

**Figure 20:**
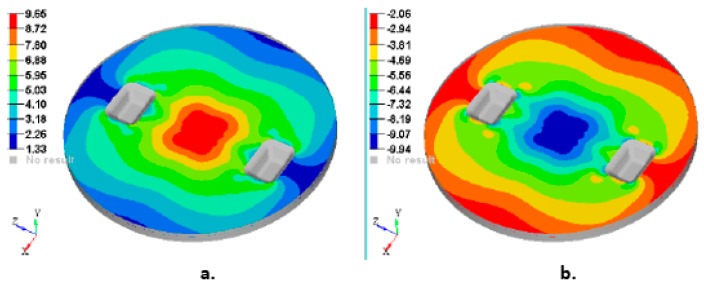
Plot of the principal stresses distribution on the manhole cover analysed: (**a**) Max. principal stresses on the lower face of the first layer; (**b**) Min. principal stresses on the upper face of the fifth layer.

Other important output parameters from the FEA analysis are: Von Mises stress (VMS), maximum and minimum Principal stress. To interpret and explain the strength of the composite manhole cover, Figure 19 and Figure 20 show the stress plot results.

A maximum of VMS of 9.66 MPa is obtained in the handling area of the manhole cover. The reason for this result could be a stress concentrator caused by the transition area from five layers to four layers because the OSB layer is missing from the sandwich structure corresponding to area C (see Figure 3).

Figure 20 shows the maximum and minimum principal stress on the lower face of the first layer and on the upper face of the fifth layer (last layer), respectively. 

### 3.3. Results Obtained from the Experimental Strain Analysis/Data of the Manhole Cover

**Figure 21:**
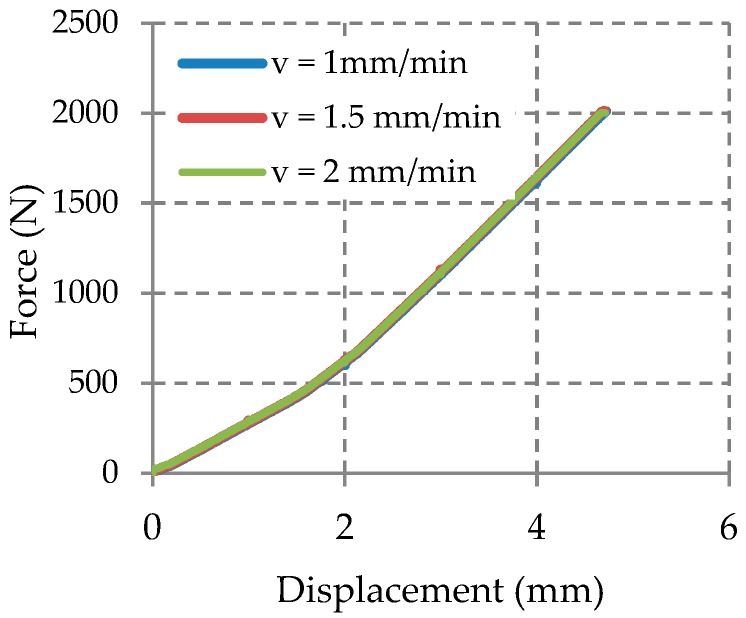
Force-displacement graph obtained on composite plate at different speeds loading.

The results obtained through the testing machine conclude that a little change of the loading speed has a little influence on the force-displacement curve (Figure 21). The experimental data shown, were recorded for the speed loading of 1 mm/min.

**Figure 22:**
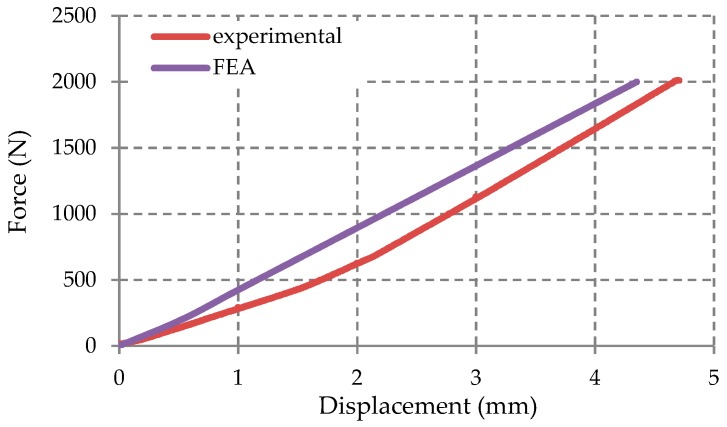
Comparison of the force-displacement curve: Experimental versus FEA.

The force–displacement curve obtained from the experimental test was compared with the force–displacement curve obtained from the FEA analysis (Figure 22). This comparison shows a good correlation between two set of results (experimental vs. FEA).

**Figure 23:**
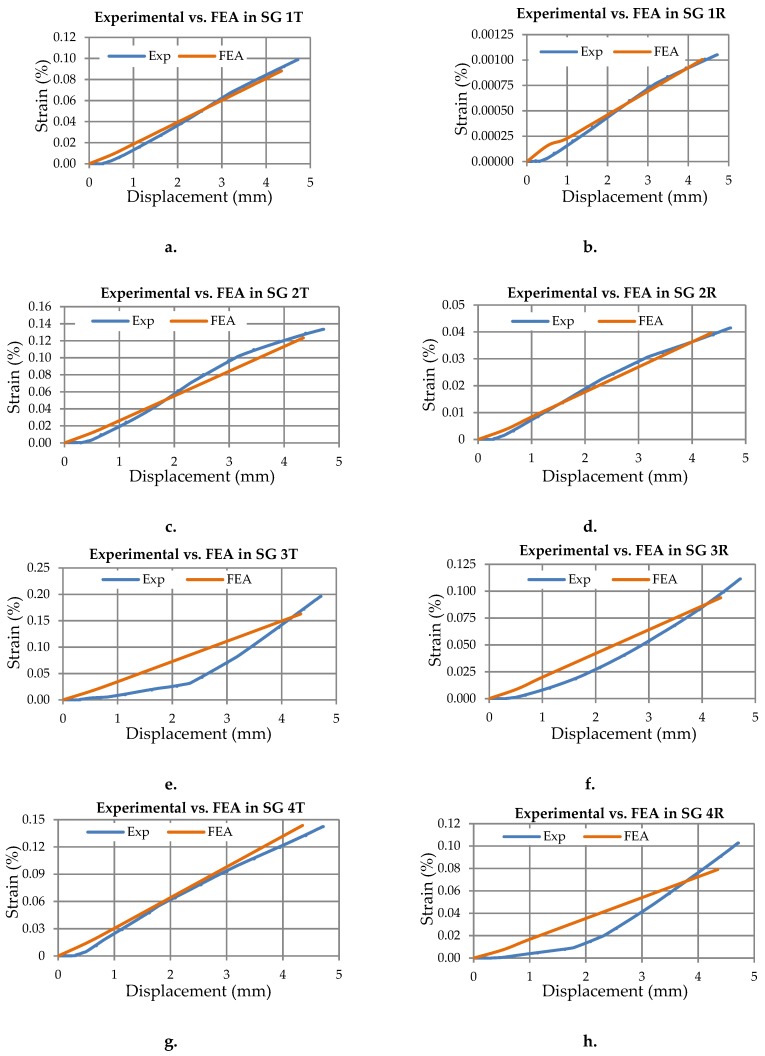

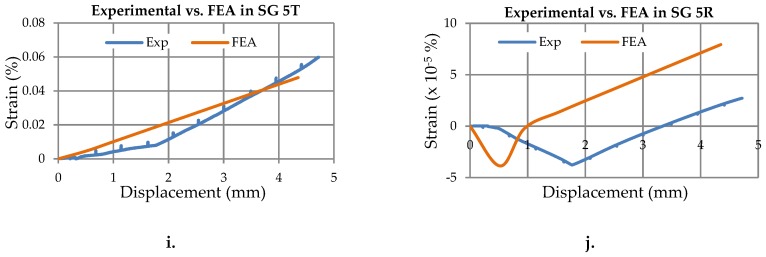
Comparison of the strain-displacement curves: Experimental versus FEA: (**a**,**b**) strain gauge (SG) no. 1 on tangential and radial directions; (**c**,**d**) strain gauge (SG) no. 2 on tangential and radial directions; (**e**,**f**) strain gauge (SG) no. 3 on tangential and radial directions; (**g**,**h**) strain gauge (SG) no. 4 on tangential and radial directions; (**i**,**j**) strain gauge (SG) no. 5 on tangential and radial directions.

To emphasize the correlation between the results obtained by the experimental test and FEA in the case of the circular composite manhole cover involved, this research shows the comparison of the strains obtained in each position of the strain gauge (Figure 23) and in each direction (radial R and tangential T directions). Thus, the strains in radial R or tangential T directions corresponding to a certain strain gauge, is plotted against the displacement recorded for the vertical force actuator.

The following abbreviations are used in the graphs shown in Figure 23: Exp-refers to curves from the experimental tests; SG–strain gauge; R-radial direction with respect to the cylindrical coordinate system whose axis passes through the centre of the circular manhole cover; T–tangential direction of the cylindrical coordinate system.

The strains recorded from all strain gauges were recorded by using the Easy Catman software compatible with the QuantumX MX840 data acquisition device and then these were compared with the values obtained from the FEA. 

The comparison between the strains experimentally obtained and the strains obtained by the FEA are shown in Table 5.

The causes of mismatch between the strains obtained by the FEA and the strains experimentally measured may include: Improper alignment of the directions corresponding to each strain gauge with respect to the radial or tangential directions of the manhole cover; anisotropy of the composite layer at the microscopic level.

## 4. Conclusions

The significant correlation between the experimental strain data and the strain results obtained by the FEA, lead to the validation of the numerical model of the composite manhole cover involved in this work. This means that the main purpose of the work was reached.

Analysing the strains measured shows small strains. Maximum values recorded are 0.00196 (0.196%) and 0.00113 (0.111%) in the tangential and radial direction respectively, of gauge 3. 

According to the FEA analysis (Figure 20a), the maximum stress obtained in the manhole cover analysed is 9.66 MPa corresponding to the MAT450 glass fiber/polyester resin. This maximum stress is much lower than 49 MPa that represents the average value of the maximum bending stress corresponding to the MAT450 glass fiber/polyester composite (Table 4).

In other words, the composite manhole cover analysed has the strength capability to be subjected to an external force that is greater than 2 kN, which gives the possibility to recommend this kind of cover for water and sewage networks or for pedestrian traffic. For implementation in city streets with heavy traffic, a more detailed analysis is necessary to determine the maximum load capability based on realistic loading data coming from vehicle traffic. 

If the manhole cover analysed in this study is intended to be applied on city streets that display heavy traffic, certain changes of the geometry or material structure of the sandwich composite are required in order to ensure that the cover can withstand the loadings.

## Figures and Tables

**Table 1:** The dimensions of the bending specimens, corresponding standards and span.

Layer No.	Composite Material	Length $L_{0}$	Width *b*	Thickness *h*	Standards	Span L
		(mm)	(mm)	(mm)		(mm)
1 & 5	MAT 450 glass fiber/polyester resin	80	10	6.5	EN ISO 14125: 1998 [20]	60
2 & 4	Chopped glass fiber fiber/polyester resin	80	10	6.5	EN ISO 14125: 1998 [20]	60
3	OSB (Oriented Strand Board)	250	50	10	EN 310: 1993 [21]	200

Dimensions $L_{0},b,h,L$ are shown in the Figure 4.

**Table 2:** Positions of the strain gauges with respect to the centre of the manhole cover.

Number Strain Gage	Distance of the Strain Gauges with Respect to the Centre (mm)
1 & 5	220
2	150
3 & 4	85

**Table 3:** The elastic properties corresponding to the layers of the manhole cover.

Property	OSB	MAT 450 Glass Fibers/Polyester Resin Composite	Chopped Glass Fibers/Polyester Resin Composite
E (MPa) *	3924	3771	861
ν	0.3	0.4	0.16

* Values corresponding to the modulus of elasticity was experimentally determined on specimens.

**Table 4:** Properties of materials corresponding to each layer of the manhole cover structure.

Material Tested	Maximum Force (N)	Flexural Modulus E (MPa)	Maximum Bending Stress (MPa)	Displacement at max. Force (mm)
MAT 450 glass fibers/polyester resin composite	216 (28)	3771 (535)	49 (5.4)	8.47 (0.64)
Chopped glass fibers/polyester resin composite	166 (20)	861 (152)	38 (4.7)	4.33 (0.54)
OSB	443 (66)	3924 (530)	25 (4.4)	5.90 (0.85)

**Table 5:** Maximum strain values obtained on the composite plate in the five locations.

Force (kN)	Displacement of the Vertical Force Actuator (mm)	Gauge Number	Strain ε (%)			
			Experimental		FEA	
			Tangential Direction	Radial Direction	Tangential Direction	Radial Direction
**2.0**	4.7	1	0.0987	0.00105	0.08816	0.00101
		2	0.1330	0.0415	0.12345	0.03956
		3	0.1960	0.1113	0.1627	0.09384
		4	0.1423	0.1026	0.1436	0.0800
		5	0.0598	2.71e-5	0.0478	7.93e-5
